# Longevity-Associated Transcription Factor *ATF7* Promotes Healthspan by Suppressing Cellular Senescence and Systematic Inflammation

**DOI:** 10.14336/AD.2022.1217

**Published:** 2023-08-01

**Authors:** Yaqun Huang, Ming-Xia Ge, Yu-Hong Li, Jing-Lin Li, Qin Yu, Fu-Hui Xiao, Hong-Shun Ao, Li-Qin Yang, Ji Li, Yonghan He, Qing-Peng Kong

**Affiliations:** ^1^State Key Laboratory of Genetic Resources and Evolution, Key Laboratory of Healthy Aging Research of Yunnan Province, Kunming Key Laboratory of Healthy Aging Study, KIZ/CUHK Joint Laboratory of Bioresources and Molecular Research in Common Diseases, Kunming Institute of Zoology, Chinese Academy of Sciences, Kunming, China.; ^2^Department of Dermatology/National Clinical Research Center for Geriatric Disorders, Xiangya Hospital, Central South University, Changsha 410000, China.; ^3^Hunan Key Laboratory of Aging Biology, Xiangya Hospital, Central South University, Changsha, China.; ^4^Kunming College of Life Science, University of Chinese Academy of Sciences, Beijing, China.; ^5^CAS Center for Excellence in Animal Evolution and Genetics, Chinese Academy of Sciences, Kunming, China.

**Keywords:** *ATF7*, cellular senescence, H3K9me2, longevity, NF-κB

## Abstract

Aging is characterized by persistent low-grade systematic inflammation, which is largely responsible for the occurrence of various age-associated diseases. We and others have previously reported that long-lived people (such as centenarians) can delay the onset of or even escape certain major age-related diseases. Here, by screening blood transcriptome and inflammatory profiles, we found that long-lived individuals had a relatively lower inflammation level (*IL6*, *TNFα*), accompanied by up-regulation of activating transcription factor 7 (*ATF7*). Interestingly, *ATF7* expression was gradually reduced during cellular senescence. Loss of *ATF7* induced cellular senescence, while overexpression delayed senescence progress and senescence-associated secretory phenotype (SASP) secretion. We showed that the anti-senescence effects of *ATF7* were achieved by inhibiting nuclear factor kappa B (NF-κB) signaling and increasing histone H3K9 dimethylation (H3K9me2). In *Caenorhabditis elegans*, *ATF7* overexpression significantly suppressed aging biomarkers and extended lifespan. Our findings suggest that *ATF7* is a longevity-promoting factor that lowers cellular senescence and inflammation in long-lived individuals.

## INTRODUCTION

Aging is a major risk factor for several diseases that limit health span, including neurodegenerative diseases, metabolic syndromes, cancer, and cardiovascular disease [1]. Persistent low-level chronic inflammation associated with aging is largely responsible for these diseases [2,3]. Aging is characterized by various molecular hallmarks, the targeting of which may accelerate or retard normal aging processes. Among them, cellular senescence is closely associated with age-related diseases, as senescent cells (SnCs) secrete a series of senescence-associated secretory phenotype (SASP) factors, resulting in persistent low-grade systemic inflammation [4,5]. Inhibiting cellular senescence and/or age-related inflammation may delay aging and slow the development of age-associated diseases. For example, selective elimination of SnCs (senolytic), or suppression of SASP secretion (senomorphic) via transgenic or pharmaceutical strategies can slow aging and age-related diseases, thereby extending healthy lifespan [6].

Although aging is an irreversible process with progressive loss of physiological integrity, impaired function, and increased vulnerability to death, some long-lived species display a delayed aging phenotype, including long-lived humans [7]. As a paradigm of successful human aging, long-lived individuals (LLIs) can reach an extremely advanced age (>90 years) without developing serious age-related diseases, thus living a healthier and longer lifespan [8]. We previously reported that LLIs exhibit enhanced autophagy-lysosomal activity [9], lower ribosomal biogenesis [10], and superior metabolic profiles [11,12], which may contribute to their healthy aging. Levels of inflammatory signaling increase during aging, which is linked to many age-related diseases [2]. However, centenarian cells show an anti-inflammatory phenotype [13]. Recently, Sayed et al. constructed an inflammatory clock of aging and found that centenarians had a lower inflammatory clock index associated with longevity [5]. In the current study, we found that LLIs displayed lower levels of proinflammatory factors such as *IL6* and *TNFα*. However, how long-lived centenarians gain these survival and healthy aging advantages remains unclear. Based on unbiased transcriptomic and interaction analyses, we identified activating transcription factor 7 (*ATF7*) as a potential contributor to the survival advantage of LLIs. ATF7 can bind to the cyclic AMP response element (CRE), which belongs to the ATF/CREB superfamily [14-16]. ATF7 activity is growth- and stress-initiated, and its transcriptional function strictly requires phosphorylation by mitogen-activated protein kinases (MAPKs) [17]. In the absence of stress, ATF7 represses the transcription of its target genes, including those encoding factors involved in innate immunity, by recruiting H3K9 di- and tri-methyltransferases G9a, EDET/SET-DB1, and Suv39h1 [18,19]. In the presence of stress, ATF7 is phosphorylated by p38MAPK, leading to the release of ATF7 and methyltransferases from target genes, thereby activating transcription [19]. During aging, the relatively preserved innate immune response overwhelms the more variable adaptive immune response, leading to higher production of proinflammatory mediators [20]. These circulating proinflammatory factors can, in turn, keep the immune system in a state of chronic low-level activation [3].

In this study, we found that relatively low levels of inflammation were accompanied by an up-regulation of *ATF7* in LLIs. Intriguingly, *ATF7* was not upregulated during the process of cellular aging, but rather was decreased. We demonstrated that *ATF7* can suppress cellular senescence and systematic inflammation by modulating nuclear factor kappa B (NF-κB) signaling and dimethylation levels of histone H3K9 (H3K9me2). We also demonstrated that *ATF7* extended the lifespan of *Caenorhabditis elegans* (*C. elegans*). These findings suggest that *ATF7* may be an important contributor to longevity by suppressing cellular aging and systematic inflammation.

## MATERIALS AND METHODS

### Study subjects, RNA sequencing (RNA-seq), network construction, and centrality analysis

As described in our prior study [10], we sampled 135 females from longevous families in Hainan Province, southern China, and constructed 76 and 59 sequencing libraries using poly(A) capture and ribosomal RNA depletion methods, respectively. The RNA-seq reads were filtered to obtain clean reads [21], aligned to the human (hg19) and *C. elegans* (WBcel235) reference genomes [22], and quantified to determine gene abundance [23]. Differentially expressed gene (DEG) analysis was performed using the ‘limma’ package [24], with the effects of library type and cell composition considered (*Gene expression ~ 0 + Group + Lymph + Neu + Mid*). The ‘removeBatchEffect’ function in ‘limma’ was used to remove the effect of library type on longevous family transcriptome data. The DESeq2 function in the R platform was used to detect DEGs between different experimental groups. A parameter false discovery rate (FDR) cutoff of less than 0.05 was considered statistically significant. Pearson correlation coefficient (r) was used to determine correlations between differently expressed transcription factors (TFs) and immune-related genes, with a Benjamini-Hochberg adjusted *P*-value cutoff of less than 0.05. The immune-related gene list was downloaded from the ImmPort database (www.immport.org/home) and TFs were obtained from the footprintDB and TRRUST databases (http://floresta.eead.csic.es/footprintdb/index.php; www.grnpedia.org/trrust/). Co-expression network plots were constructed using Cytoscape (v3.9.0) [25] and centrality analysis was conducted using the cytoHubba program in Cytoscape. Functional enrichment analysis was performed using “Metascape” [26] and “DAVID” [27,28]. Raw RNA-seq data of replicative senescent human embryonic lung fibroblasts (WI-38), human neonatal foreskin fibroblasts (HFF and BJ), and oncogene-induced senescent human embryonic lung fibroblasts (IMR-90) were collected from the ‘‘GEO Repository’’ (www.ncbi.nlm.nih.gov/geo/) (accession numbers GSE63577, GSE64553, GSE61130).

### Cell culture

Human dermal fibroblasts (HDFs) were obtained from circumcised foreskins of healthy human donors aged 5-20 years in Xiangya Hospital, Central South University, as described previously [29,30]. Human embryonic lung fibroblasts IMR-90 and HEL were purchased from the American Type Culture Collection (ATCC) and Kunming Cell Bank (Chinese Academy of Sciences), respectively. The HDF, HEL, and IMR-90 cells were cultured in Dulbecco’s Modified Eagle Medium (DMEM, C11995500BT, Gibco, USA) or Minimum Essential Medium (MEM, C11095500BT, Gibco, USA) supplemented with 10% fetal bovine serum (35-076-CV, Gibco, USA) and 1% penicillin/streptomycin (15140-122, Gibco, USA) in a 37 °C/5% CO_2_ humidified incubator. Human embryonic kidney HEK-293T cells were purchased from ATCC and cultured in DMEM (C11995500BT, Gibco, USA) supplemented with 10% fetal bovine serum (04-001-1ACS, BI, Israel) and 1% penicillin/streptomycin (15140-122, Gibco, USA) in a 37 °C/5% CO_2_ humidified incubator.

## Plasmids and lentiviral infection

*ATF7* overexpression plasmid was constructed using the pCDH-MSCV-E2F-eGFP lentiviral vector. *ATF7* short hairpin RNA (shRNA) (target sequence: CGAAGAA CTCACTTCTCAGAA; CCGAACTGACTCAGTCATC AT) plasmids were constructed using the pLKO.1 lentiviral vector. Plasmids were transfected into HEK-293T cells, with the collected lentiviruses then transduced into fibroblasts (HDF, IMR-90). Cells were subsequently cultured in selected media supplemented with 2 μg/mL puromycin (ant-pr-1, InvivoGen, USA).

### Senescence-associated beta-galactosidase (SA-β-gal) staining

SA-β-gal staining was performed using a Senescence Cells Histochemical Staining Kit (CS0030-1kt, Sigma-Aldrich, USA) according to the manufacturer’s instructions. Briefly, cells were washed with phosphate-buffered saline (PBS) and fixed at room temperature for 7 min, then incubated in staining working solution overnight at 37 °C. Cells were visualized using a Nikon eclipse Ti inverted microscope and a minimum of 200 cells were counted.

### Quantitative real-time polymerase chain reaction (qRT-PCR)

Total RNA was extracted using TRIzol reagent (15596018, Invitrogen, USA) and reverse transcribed into cDNA using a RevertAid First Strand cDNA Synthesis Kit (K1622, Thermo Fisher Scientific, USA) according to the manufacturer’s protocols. qRT-PCR was performed using 2 × Tsingke® Master qPCR Mix (TSE201, Tsingke, China), with actin as the endogenous normalization control. Gene expression levels were calculated using the 2^-ΔΔCt^ method [31]. All primers used are presented in Supplementary Table 1.

### Western blotting

Cells were lysed using RIPA lysis buffer (P0013B, Beyotime, China) supplemented with 1% phenylmethylsulfonyl fluoride (ST506, Beyotime, China) on ice. Protein extract (30 μg) was separated by sodium dodecyl-sulfate polyacrylamide gel electrophoresis (SDS-PAGE) and subsequently transferred to polyvinylidene fluoride (PVDF) membranes (162-0177, Bio-Rad, USA). The membranes were incubated with appropriate primary antibodies at 4 °C overnight, then with horseradish peroxidase (HRP)-labeled goat anti-rabbit or anti-mouse immunoglobulin G (IgG) (H + L) secondary antibodies at room temperature for 1 h. Protein bands were visualized using an ECL Detection Kit (UEL-S6009L, US Everbright, China). Quantification of blots was performed using ImageJ software. Detailed information on primary antibodies used in western blotting is provided in Supplementary Table 2.

### EdU incorporation assay

Cell proliferation was analyzed using a Cell-Light EdU Apollo567 *In Vitro* Kit (C10310-1, Ribobio, China). Briefly, cells (8 × 10^3^) were seeded into each well of a 96-well plate and incubated for 24 h with 5 μM EdU at 37 °C. The cells were then added with 1 × reaction buffer for 30 min. Images were captured using a Cytation 5 Cell Imaging Multi-Mode Reader (BioTek).

### Cellular reactive oxygen species (ROS) detection

Cellular ROS detection was performed using CellROX® Oxidative Stress Reagent (C10422, Thermo Fisher Scientific, USA) according to the manufacturer’s instructions. Briefly, cells (8 × 10^3^) were seeded into each well of a 96-well plate, followed by the addition of CellROX® Reagent at a final concentration of 10 μM and incubation for 30 min at 37 °C. Images were captured using a Cytation 5 Cell Imaging Multi-Mode Reader (BioTek). Quantification was performed using ImageJ software.

### Cytokine array

Cells were seeded into 6-cm dishes and washed and incubated in serum-free DMEM in a 37 °C/5% CO_2_ humidified incubator. After 24 h, the conditioned medium (CM) was collected, filtered (0.2 μm), and frozen at -80 °C. The CM was analyzed using an antibody array (QAH-CYT-1, RayBiotech, USA) according to the manufacturer’s instructions. The array was used to detect 20 cytokines and chemokines, including IL1A, IL1B, IL2, IL4, IL5, IL6, IL8 (CXCL8), IL10, IL12, IL13, IFNγ, TNFα, GM-CSF, GROα/β/γ, MCP1 (CCL2), MIP1a (CCL3), MIP1b (CCL4), MMP9, VEGFA, and RANTES (CCL5). The quantity of secreted cytokines was measured by adding 90 μL of concentrated conditioned medium to the array, followed by overnight incubation at 4 °C. After washing with wash buffer, biotinylated primary antibodies were added and incubated at room temperature for 2 h. After washing again with wash buffer, Cy3 equivalent dye-conjugated streptavidin was added for 1 h. The chip was scanned using an InnoScan 300 Microarray Scanner (Innopsys, France). Cytokines were quantified according to the standard curve calibrated from the same array.

### Chromatin immunoprecipitation (ChIP) analysis

ChIP analysis was performed using a SimpleChIP® Enzymatic Chromatin IP Kit (9003, Cell Signaling Technology, USA) according to the manufacturer’s instructions. Chromatin was crosslinked with 1% formaldehyde in cell medium at room temperature for 10 min. Glycine was then added to the medium to terminate the reaction. After washing in ice-cold PBS, cells were lysed and sonicated into 150-900-bp fragments. Immunoprecipitation was performed overnight at 4 °C with anti-histone H3K9me2 and anti-histone H3. Normal rabbit IgG was used as a negative control. The immunocomplexes were washed using ChIP-Grade Protein G Magnetic Beads (9006, Cell Signaling Technology, USA) and incubated at 65 °C overnight following the addition of 5 M NaCl and Proteinase K to reverse the crosslinks. Free precipitated DNA was further purified. The DNA samples were used for qRT-PCR. All primers are presented in Supplementary Table 3.

### Co-immunoprecipitation

The HDF cells were lysed in IP-lysis buffer (50 mM Tris-HCl, 150 nM NaCl, 1 mM ethylenediaminetetraacetic acid, 0.2% NP40, and 10% glycerol) for 30 min on ice. After centrifugation at 12 500 rpm and 4 °C for 15 min, the supernatants were incubated in anti-ATF7 or control IgG. The immunocomplexes were then collected using Protein-A-agarose beads (11134515001, Roche, Switzerland) and subjected to western blotting with anti-G9a antibodies.

### Cleavage Under Targets and Tagmentation (CUT&Tag) assay

CUT&Tag assay was performed using a Hyperactive^®^ Universal CUT&Tag Assay Kit for Illumina (TD903, Vazyme, China) according to the manufacturer’s instructions. Briefly, 1 × 10^5^ cells were collected, washed in wash buffer, and added to 10 μL of activated ConA Beads. Bead-bound cells were incubated overnight at 4 °C with anti-histone H3K9me2. Normal rabbit IgG was used as a negative control. The cells were washed and incubated with rabbit secondary antibodies at room temperature for 1 h. After washing, the cells were incubated with pA/G-Tnp at room temperature for 1 h, then with TruePrep Tagment Buffer L (TTBL) and Dig-300 buffer for fragmentation at 37 °C for 1 h. The released chromatin fragments were extracted, and libraries were generated using TruePrep® Index Kit V2 for Illumina (TD202, Vazyme, China). Reads were aligned to the human reference genome hg19 using Bowtie2 (v.2.4.1) [32]. The ‘picard.jar’ program in GATK (v4.1.8.0) was used to remove duplicated reads [33]. For each replicate, peaks were first called using MACS2 (v.2.1.2) [34] with the parameters: -q 0.05 -B. Differential binding analysis of peaks was performed using the Bioconductor package ‘DiffBind’. The ‘annotatedPeak’ function in the R package ‘CHIPseeker’ [35] was used to annotate peaks. Peaks were visualized using Integrative Genomics Viewer (IGV) [36].

### Lifespan assay

All *C. elegans* lifespan experiments were conducted at 20 °C in accordance with standard protocols using HT115/RNA interference (RNAi) (with addition of Amp). Briefly, worms were branched to obtain synchronized eggs, which were cultured to hatch and used for lifespan assays. At L4 molting, animals were transferred to plates containing 20 M 5-fluoro-2’-deoxyuridine (FUDR), which kills their progeny at the embryo stage but does not significantly affect lifespan. The plates were refreshed daily. Approximately 50 worms were used for each treatment. The first day of adulthood was recorded as day 1 and dead worms were counted from day 5. Worms that did not respond to nose touch were picked out of the bacterial lawn, and worms that did not move were scored as dead.

### Body bending assay

Worms were pretreated as in the lifespan assays. On the day of examination, 100 μL of M9 buffer was pipetted onto the surface of an empty 35-mm NGM plate. A single worm was added to the buffer and allowed 30 s to recover from the transfer. The number of thrashing movements over a 30-s period was counted (four times per worm, 15-20 worms per treatment). One worm from one treatment was counted, then one worm from another treatment was counted, and so on, with the cycle repeated to avoid environmental or operational interference.

### Lipofuscin accumulation

Autofluorescence of intestinal lipofuscin was measured as an index of senescence in day 10 adults. Worms were randomly selected from each bacterial lawn and washed three times with M9 buffer. The worms were then placed on 5% agar pads coated with 10 mM sodium azide in M9 buffer to initiate paralysis. Lipofuscin autofluorescence images were taken using blue excitation light (405-488 nm) with 4’,6-diamidino-2-phenylindole (DAPI) and laser confocal scanning microscopy. Fluorescence was quantified using ImageJ software to determine lipofuscin levels. Three independent experiments were performed with more than 30 worms for each bacterial species each day.

### Pharyngeal pumping

Wild-type and *ATF7*-overexpressing worms were synchronized by timed oviposition onto an empty vector (control) and *ATF7* double-stranded RNA (dsRNA)-expressing HT115. Day 1 adult worms were transferred to control and RNAi plates with FUDR. Pumping rates were determined on day 10 of adulthood. Terminal pharyngeal bulb pumps were counted at 30-s intervals for each worm on the bacterial lawn. The pumping rate per minute was averaged for 10 worms per treatment. Pumping rates were assessed in three independent replicate experiments.

### RNAi in C. elegans

RNAi *ATF7* was generated using primers GAATTCC TGCAGCCCCGCGTCCAATTGATGGGC and ACGC GTGGATCCCCCCACAGATAACAATTTGGAAATGTCC and linked to L4440 by infusion (SmaI). The *E. coli* strain HT115 (DE3) expressing *ATF7* dsRNA was grown overnight at 37 °C in Luria-Bertani (LB) broth containing 100 μg/mL ampicillin, then spread on nematode growth medium (NGM) plates containing 100 μg/mL ampicillin and 1 mM isopropyl 1-thio-β-D-galactopyranoside (IPTG). The RNAi-expressing bacteria were then grown at 25 °C overnight. Synchronized L4 larvae were placed on the plates at 20 °C. RNAi was induced from L4 unless otherwise noted. L4 adult animals were used for further experiments.

### ATF7 overexpression in C. elegans

*ATF7* cDNA expression was driven by an *ATF7* promoter and *ATF7* cDNA insertion was verified by sequencing. The primers used for cloning *ATF7* were: *ATF7* promoter-F: GTCGACTCTAGAGGATCCCCTT TTGGAGCCG CCAAAGATCG; *ATF7* promoter-R: CGACCGACATA ATTGTGTTGGACGATTGCTGA; *ATF7* cDNA -F: CAACACAATTATGTCGGTCGTAACAACGA; *ATF7* cDNA-R: TGGGTCCTTTGGCCAATCCCTTGGAGT TT GGGTAATTTCAGTTGC. The cDNA and promoter of *ATF7* were then linked to pPD95_75 by infusion (SmaI). The plasmid was injected at a concentration of 50 ng/μL into N2 worms using a Pmyo-3::mCherry::unc-54 (pCFJ104) expressing plasmid as a co-injection marker.

### Statistical analysis

Statistical analyses were conducted using GraphPad Prism v9 (GraphPad Software). Data are presented as means ± standard error of the mean (SEM). Normal distribution of data was determined using the Shapiro-Wilk test. Comparisons were performed using two-tailed Student’s *t*-test or Mann-Whitney U test. Survival curves were compared, and *P*-values were calculated using log-rank (Mantel-Cox) analysis. *P* < 0.05 was defined as statistically significant.

## RESULTS

### Centenarians display lower inflammation with possible ATF7 involvement

**Figure 1.**
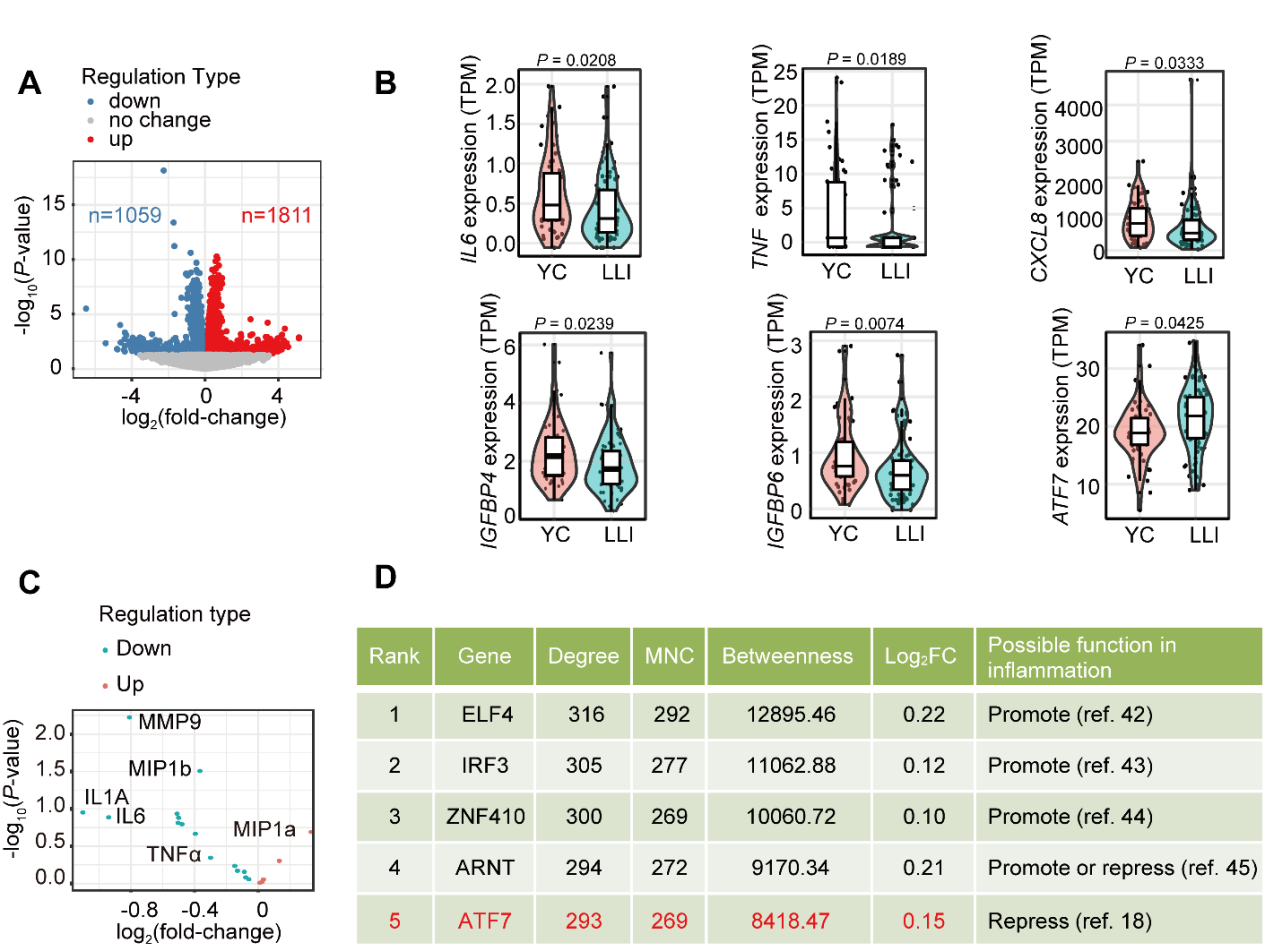
**Identification of *ATF7* in DEGs between LLIs and YCs**. (**A**) Volcano plot of DEGs identified based on *P*-value and fold-change of genes with expression differences between all LLI and YC transcripts (n = 80, 55). (**B**) Expression of representative pro-inflammatory factor and *ATF7* expression levels in LLI and YC transcripts (n = 80 and 55 for LLI and YC, respectively). (**C**) Proteins in plasma collected from LLIs and YCs, measured by cytokine array (n = 24 and 25 for LLI and YC, respectively). (**D**) Rank of local- and global-based method values of candidate TF genes. LLIs: long-lived individuals; YCs: younger controls; MNC, Maximum neighborhood component; FC, fold-change.

Peripheral blood mononuclear cells (PBMCs) from centenarians show reduced *IL6* mRNA levels [13]. To determine overall gene expression changes in proinflammatory factors in LLIs, we analyzed RNA-seq data in a cohort of longevous families [10]. In total, 2 870 significant DEGs were identified between LLIs and younger controls (YCs) (*P* < 0.05) (Fig. 1A). Among the DEGs, a portion of major proinflammatory factors, including *IL6*, *CXCL8 (IL8)*, *TNF*, *IGFBP4*, and *IGFBP6* [37-39], were down-regulated in LLIs compared to YCs (Fig. 1B). Consistent with the RNA-seq results, cytokine array showed that LLIs had significantly lower levels of MMP9 and CCL4 (MIP1b) and lower levels of proinflammatory factors, including IL6, IL1A, and TNFα, compared to the sex-matched YCs (Fig. 1C). As proinflammatory factors are primarily regulated at the transcriptional level, we focused on differentially expressed TF genes that may have both anti-inflammatory and potentially anti-aging functions. We performed network centrality analysis to prioritize candidate TF genes [40,41], and identified five TF genes related to inflammatory regulation (Fig. 1D). Among them, *ELF1*, *IRF3*, *ZNF410*, and *ARNT* may promote the development of inflammation or senescence [42-45]. *ATF7* not only plays a repressive role in innate immunity [18], but also responds to intrinsic and extrinsic stresses [17], and thus was chosen as the candidate gene (Fig. 1B, D). We confirmed the expression level of *ATF7* in LLIs and YCs using qRT-PCR (Supplementary Fig. 1A) and explored its potential roles and mechanisms in the following experiments.

### Manipulation of ATF7 alters cellular senescence progression

**Figure 2.**
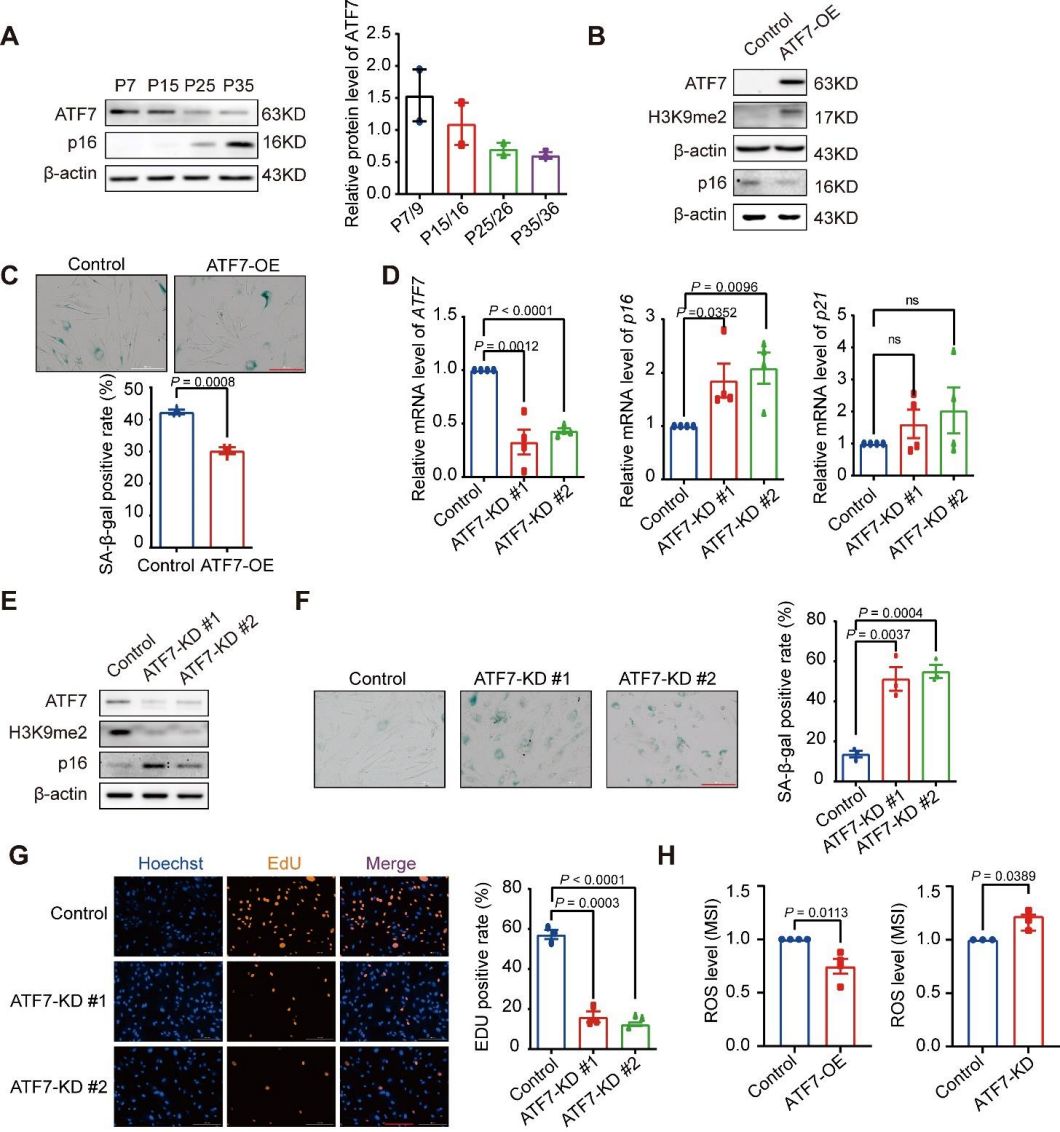
**Manipulation of *ATF7* expression changes cellular senescence progression in HDFs**. (**A**) ATF7 protein levels were assessed during serial passage by western blotting (n = 2 biological replicates). (**B**) Western blot analysis of protein levels of ATF7, p16, H3K9me2 in control and *ATF7*-overexpressing HDFs (n = 3 biological replicates). (**C**) SA-β-Gal-staining cells in control and *ATF7*-overexpressing HDFs. Typical images are presented on top and quantitative result is presented at bottom (n = 3 biological replicates, scale bar, 200 μm). (**D**) qRT-PCR analysis of mRNA levels of *ATF7*, *p21*, and *p16* in control and *ATF7*-knockdown HDFs (n = 4 biological replicates). (**E**) Western blot analysis of protein levels of ATF7, p16, and H3K9me2 in control and *ATF7*-knockdown HDFs (n = 3 biological replicates). (**F**) SA-β-Gal-staining cells in control and *ATF7*-knockdown HDFs. Typical images are presented on the left and quantitative result is presented on the right (n = 3 biological replicates, scale bar, 200 μm). (**G**) EdU-staining in control and *ATF7*-knockdown HDFs. Typical images are presented on the left and quantitative result is presented on the right (n = 3 biological replicates, scale bar, 200 μm). (**H**) Cellular ROS detected in control and *ATF7*-overexpressing (left, n = 4 biological replicates) or knockdown (right, n = 3 biological replicates) HDFs. P, passage; OE, overexpression; KD, knockdown; MSI, Mean signal intensity. Statistical analyses were performed using two-sided Student’s *t*-test.

As *ATF7* was upregulated in LLIs compared to YCs, we investigated whether it affects cellular senescence, one of the major hallmarks of aging. Unlike the case in LLIs, the protein level of ATF7 was progressively down-regulated during serial passage in HDF cells, accompanied by the up-regulation of p16 (CDKN2A), a marker of cellular senescence (Fig. 2A). Similarly, *ATF7* was down-regulated in senescent WI-38 fibroblasts, HFF and BJ fibroblasts, and IMR-90 fibroblasts compared to non-senescent cells (Supplementary Fig. 1B) (online published data) [46-48]. The downregulation of ATF7 was partly due to increased phosphorylation by p38MAPK (Supplementary Fig. 2A), which can cause instability and degradation [49,50]. Notably, we found that application of SB203580, an inhibitor of p38MAPK, decreased the degradation of the ATF7 protein (Supplementary Fig. 2B).

**Figure 3.**
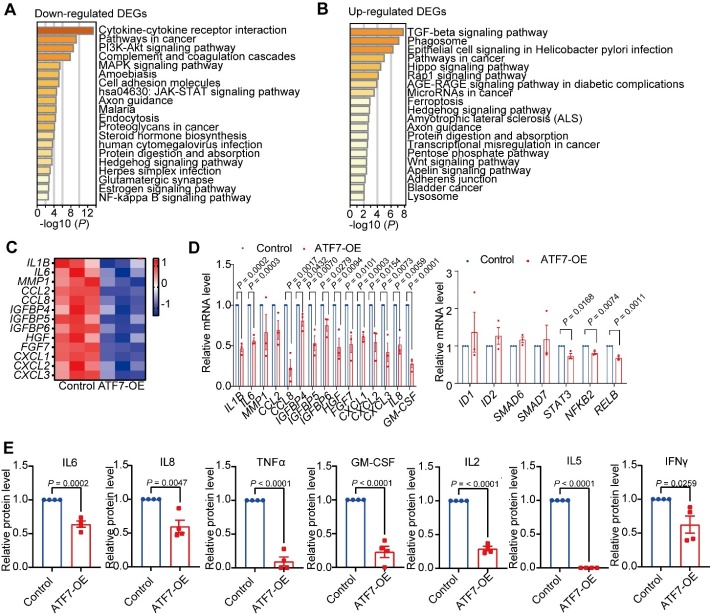
***ATF7* represses SASP secretion in HDFs**. (**A**) KEGG pathway enrichment analysis of down-regulated DEGs between control and *ATF7*-overexpressing HDFs. (**B**) KEGG pathway enrichment analysis of up-regulated DEGs between control and *ATF7*-overexpressing HDFs. (**C**) Heatmap of SASP genes for *ATF7*-overexpressing HDFs relative to the control group from RNA-seq datasets (n = 3 biological replicates). (**D**) qRT-PCR analysis of mRNA levels of SASP genes and inflammatory pathway genes in *ATF7*-overexpressing HDFs relative to the control (n = 3 biological replicates). (**E**) SASP and inflammatory factors in serum-free CM collected from control and *ATF7*-overexpressing HDFs, measured by cytokine array (n = 4 biological replicates). OE, overexpression. Statistical analyses were performed using two-sided Student’s *t*-test.

Next, we examined the effect of *ATF7* on HDF senescence. Overexpression of *ATF7* significantly decreased senescence markers, including p16 (CDKN2A) protein levels and SA-β-gal-staining positive cells, and increased H3K9me2 levels compared to control HDFs (Fig. 2B-C). In contrast, knockdown of *ATF7* with shRNA reversed the above senescence markers (Fig. 2D-F) and inhibited DNA synthesis indicated by EdU staining (Fig. 2G). These results were validated in the IMR-90 fibroblasts (Supplementary Fig. 3A-B), suggesting a suppressive role of *ATF7* in cellular senescence. As DNA damage and ROS are primarily responsible for cellular senescence, we tested ROS levels in *ATF7*-overexpressed or down-regulated cells. As shown in Fig. 2H, compared to untreated control HDFs, overexpression and down-regulation of *ATF7* significantly decreased and increased ROS levels in HDFs, respectively.

### ATF7 represses secretion of SASP

SnCs affect surrounding cells/tissues by secreting SASP, which consists of inflammatory factors, extracellular modifiers, and growth factors [51]. To test whether *ATF7* regulates SASP and to identify the pathways involved in senescence following *ATF7* manipulation, we performed transcriptome analysis of *ATF7*-overexpressing and control HDFs. In total, 827 DEGs were identified, including 534 down-regulated and 293 up-regulated DEGs in the *ATF7*-overexpressing HDFs relative to the control cells. KEGG pathway enrichment analysis indicated that the down-regulated DEGs were enriched in inflammatory pathways, including cytokine-cytokine receptor interaction, PI3K-Akt signaling pathway, MAPK signaling pathway, JAK-STAT signaling pathway, and NF-κB signaling pathway (Fig. 3A). Up-regulated DEGs were enriched in the TGF-β signaling pathway (Fig. 3B), among which *ID1*, *ID2*, *ID3*, *ID4*, *SMAD6*, *SMAD7*, and *SMAD9* are negative regulators of inflammation [52-55]. These findings suggest that *ATF7* may play an inhibitory role in modulating HDF inflammation. SnC-secreted SASP is responsible for multiple age-related pathologies [56]. Based on transcriptome data, 13 down-regulated SASP factors, together with upstream inflammation-regulatory genes (e.g., *STAT3*, *NFKB2*, and *RELB*), were confirmed using qRT-PCR (Fig. 3C, D). In addition, *ATF7* overexpression significantly reduced the mRNA expression levels of *IL8* and *GM-CSF* (Fig. 3D). In contrast, compared to the control cells, knockdown of *ATF7* up-regulated several SASP factors and down-regulated genes in the TGF-β signaling pathway, i.e., *ID1*, *ID2*, *SMAD6*, and *SMAD7* (Supplementary Fig. S4A-C). To verify the inhibitory role of *ATF7* on SASP, we analyzed SASP release in culture medium using antibody arrays. As shown in Fig. 3E, a panel of inflammatory factors, including IL6, IL8, TNFα, GM-CSF, IL2, IL5, and IFNγ, were significantly decreased in *ATF7*-overexpressing HDFs compared to the controls.

**Figure 4.**
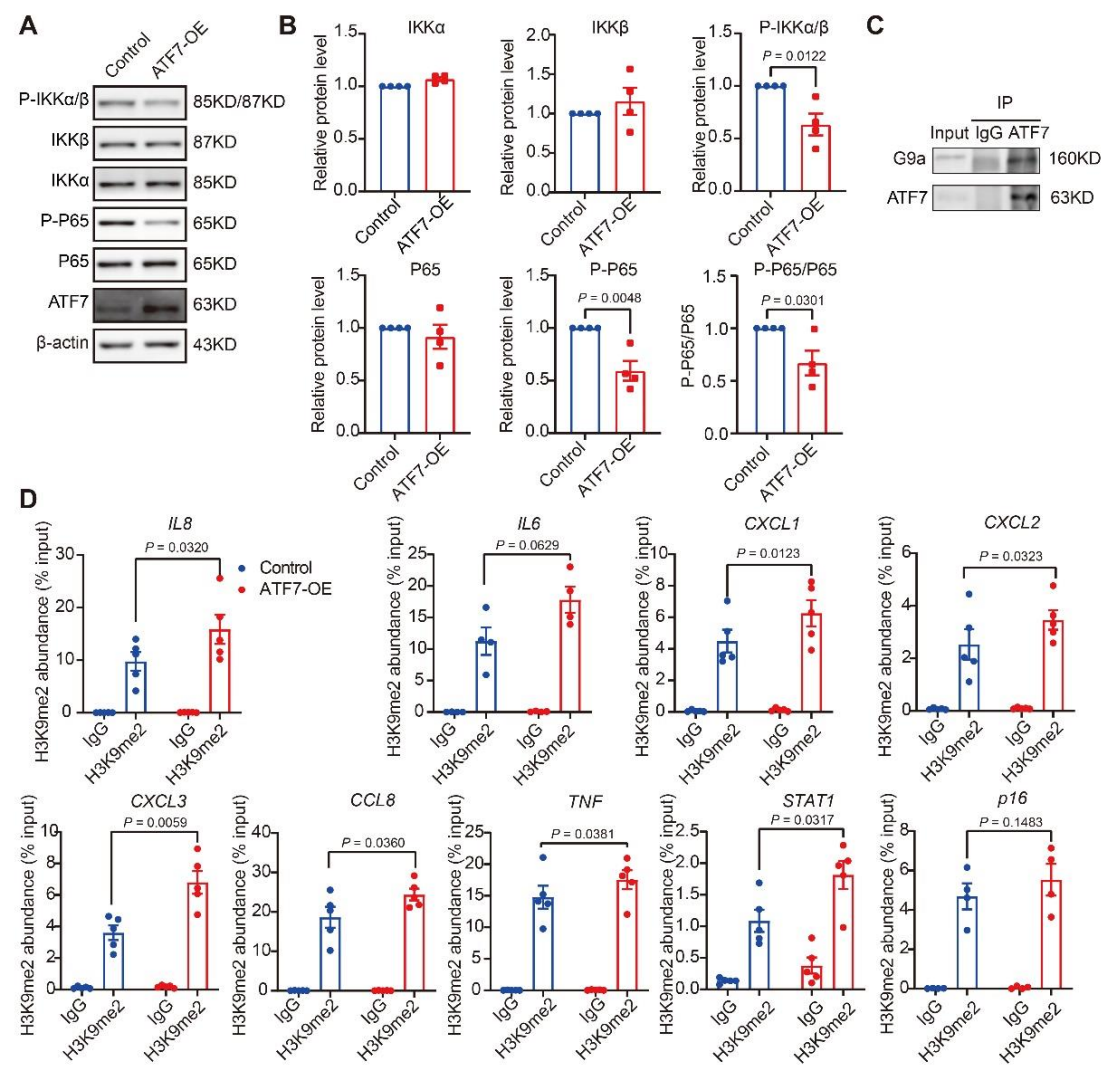
***ATF7* inhibits SASP secretion by inhibiting NF-κB signaling and modulating H3K9me2 in HDFs**. (**A**) Western blot analysis of NF-κB signaling pathway activation in control and *ATF7*-overexpressing HDFs (n = 4 biological replicates). (**B**) Qualification results of (A). (**C**) Co-immunoprecipitation of ATF7 and G9a. Cell lysates of HDFs were immunoprecipitated with anti-ATF7 antibody or control IgG, with immunocomplexes then subjected to western blotting using G9a (n = 2 biological replicates). (**D**) ChIP assay with qRT-PCR detection of H3K9me2 abundance in SASP genes at ATF7-binding sites of promoter regions in control and *ATF7*-overexpressing HDFs (n = 4 biological replicates for *IL6* and *p16*, n = 5 biological replicates for other genes). OE, overexpression. Statistical analyses were performed using two-sided Student’s *t*-test.

Notably, the release of TNFα, GM-CSF, and IL2 was markedly inhibited (>50% reduction) and IL5 was completely inhibited in the HDFs with *ATF7* overexpression relative to the control cells (Fig. 3E). The effect of *ATF7* on suppressing SASP in SnCs mimicked that in LLIs, in which *ATF7* up-regulation was accompanied by lower levels of SASP factors compared to YCs (Fig. 1B-C). These findings suggest that *ATF7* can repress cellular senescence and SASP secretion in SnCs and may contribute, at least partially, to lower inflammation in LLIs.

**Figure 5.**
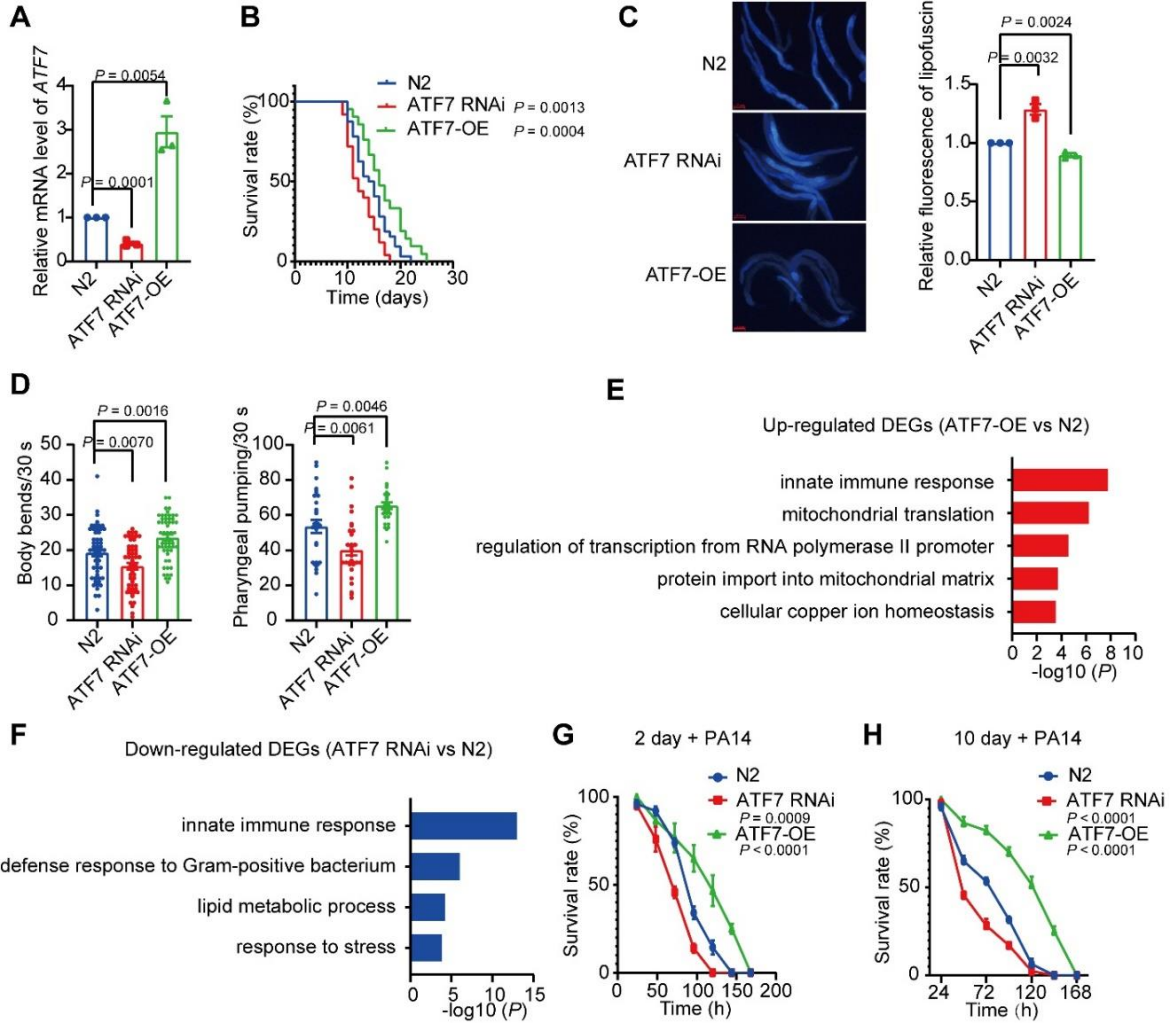
**Manipulation of *ATF7* affects lifespan and survival in *C. elegans***. (**A**) qRT-PCR analysis of *ATF7* mRNA levels in N2 and *ATF7*-overexpressing or RNAi worms (n = 3 biological replicates). (**B**) Lifespan analysis of N2 and *ATF7*-overexpressing or RNAi worms from (A) (n = 3 biological replicates). (**C, D**) Lipofuscin accumulation assay (C, n = 3 biological replicates, scale bar, 100 μm), body bending assay (D, left, n = 53/group for 3 biological replicates), and pharyngeal pumping assay (D, right, n = 30/group for 3 biological replicates) in N2 and *ATF7*-overexpressing or RNAi worms. (**E**) Gene Ontology (GO) enrichment analysis of down-regulated DEGs between N2 and *ATF7* overexpressing worms. (**F**) GO enrichment analysis of up-regulated DEGs between N2 and *ATF7*-RNAi worms. (**G**) (H) Survival analysis of N2 and *ATF7*-overexpressing or RNAi worms exposed to *P. aeruginosa* PA14. Worms were fed with PA14 for 2 days (g) or 10 days (h) after reaching adulthood (n = 3 biological replicates/group). OE, overexpression. Statistical analyses were performed using two-sided Student’s *t*-test.

### ATF7 restrains SASP by inhibiting NF-κB signaling and modulating H3K9me2

To determine how *ATF7* inhibits SASP, we focused on the inflammatory pathways enriched in KEGG analysis. Based on immunoblotting, we found that the phosphorylation level of P65, a canonical indicator of NF-κB signaling activity, was significantly decreased in *ATF7*-overexpressing HDFs compared to control HDFs (Supplementary Fig. S5A). NF-κB is a recognized regulator of SASP production [6,57]. Therefore, we investigated upstream signals in this pathway and found that the phosphorylation levels of P65 and IKKα/β were diminished in *ATF7*-overexpressed cells compared to the control cells, whereas the total protein levels of P65 and IKKα/β remained unchanged (Fig. 4A, B).

In addition to its role in regulating NF-κB signaling, *ATF7* is also reported to regulate inflammatory gene expression epigenetically [18,50]. As shown in Fig. 2B, *ATF7* overexpression up-regulated H3K9me2, which negatively regulates SASP factors *IL6* and *IL8* [58]. To investigate whether *ATF7* regulates SASP through H3K9me2, we performed chromatin immunoprecipitation plus qRT-PCR analysis using H3K9me2 antibodies. As shown in Fig. 4C and Supplementary Fig. 5B, co-immunoprecipitation revealed that ATF7 could directly bind G9a, which regulates H3K9 dimethylation. These results suggest that ATF7 may recruit G9a to regulate H3K9me2 and silence its target genes, as reported previously [18,50]. The ChIP-qRT-PCR analysis also showed that *ATF7* overexpression up-regulated H3K9me2 levels around the promotor regions of several SASP factors, including *IL8*, *IL6*, *CXCL1*, *CXCL2*, *CXCL3*, *CCL8*, and *TNF*, as well as the upstream regulator *STAT1*, but not p16 (Fig. 4D). These data were validated using the CUT&Tag assay, which showed a high signal-to-noise ratio (Supplementary Fig. 6) [59]. Thus, these results suggest that *ATF7* suppresses SASP via both NF-κB and H3K9me2.

### Manipulation of ATF7 affects lifespan and survival in C. elegans

As overexpression of *ATF7* delayed cellular senescence, suppressed SASP production *in vitro*, and was associated with longevity in LLIs, we wondered whether it may function to promote lifespan. We tested this using *C. elegans*, a model species widely used to determine molecular mechanisms of lifespan [60]. As shown in Fig. 5, transgenic *ATF7* overexpression extended the *C. elegans* lifespan by 10.3% compared to the control, whereas RNAi knockdown of *ATF7* decreased lifespan by 17.2% (Fig. 5B). Consistently, compared to the control, overexpression of *ATF7* reduced intestinal lipofuscin and promoted body locomotion and pharyngeal pumping (Fig. 5C, D), applied as aging markers in *C. elegans* [61]. In contrast, knockdown of *ATF7* accelerated aging in *C. elegans* compared to the control group (Fig. 5C, D).

To explore how *ATF7* regulates lifespan and aging in *C. elegans*, we performed RNA-seq analysis of *ATF7*-overexpressed or silenced *C. elegans* and control *C. elegans*. Gene Ontology (GO) enrichment analysis showed that the up-regulated genes in *ATF7*-overexpressed *C. elegans* relative to control *C. elegans* were mainly enriched in innate immune response, whereas the down-regulated genes in *ATF7*-silenced *C. elegans* were mainly enriched in innate immune response and defense responses against gram-positive bacteria (Fig. 5E, F). The downregulation and up-regulation of innate immune-related genes in *ATF7*-silenced and overexpressing worms support the regulatory role of *ATF7* in innate immunity [18,62,63]. Moreover, most up-regulated DEG orthologs in humans play roles in modulating immune function, depending on different physiological/pathophysiological contexts (Supplementary Table 4). Importantly, as *ATF7* is closely associated with innate immunity, we suspect it may influence the survival of pathogen-challenged *C. elegans*. Indeed, in the presence of *Pseudomonas aeruginosa* (PA14), transgenic *ATF7* overexpression enhanced *C. elegans* survival, whereas RNAi knockdown of *ATF7* reduced survival (Fig. 5G, H). Overall, these results suggest that *ATF7* can extend the lifespan and promote healthy aging in *C. elegans*, in part by regulating innate immunity genes.

## DISCUSSION

Persistent systemic chronic inflammation can lead to a series of age-related diseases [2,3]. Inflammaging is a well-recognized source of age-related inflammation [3]. As an important contributor to inflammaging *in vitro* and *in vivo*, SnC-secreted SASP can cause several age-related diseases by maintaining a proinflammatory internal microenvironment [64]. Accumulating evidence suggests that attenuating SASP production and age-related inflammation can alleviate age-related diseases and extend healthspan [2,65-67]. Our previous study and others have shown that LLIs can delay (or avoid) the onset of major age-related diseases [68-71]. We therefore wondered whether this survival advantage is achieved by maintaining better levels of inflammation. Here, by screening a panel of inflammatory factors, we found relatively low levels of inflammation in our longevity cohort. We also identified the *ATF7* transcriptional factor as a key upstream regulator in maintaining lower inflammation in LLIs. Importantly, manipulation of *ATF7* modulated cellular senescence and *C. elegans* lifespan, suggesting it may be a novel and conserved contributor for longevity across various species, including humans.

In the current study, *ATF7* was highly expressed in LLIs, but its expression was gradually lost during cellular senescence, suggesting it may be a longevity-associated factor rather than an aging-related factor. As a transcription repressor, ATF7 is involved in the regulation of cell cycle progression [72,73], heterochromatin formation [16,18,74], telomere shortening [49,75], and innate immunity [18,62,63,76]. Our results showed that overexpression of *ATF7* suppressed cellular senescence and various inflammatory factors, indicating a possible role in delaying aging in individuals. Indeed, *ATF7* has been reported to affect aging in mice, with its depletion found to promote senescence of embryonic fibroblasts and shorten lifespan [77]. Here, we provide evidence that *ATF7* acts as a longevity-promoting factor in humans by inhibiting cellular senescence and SASP secretion.

**Figure 6.**
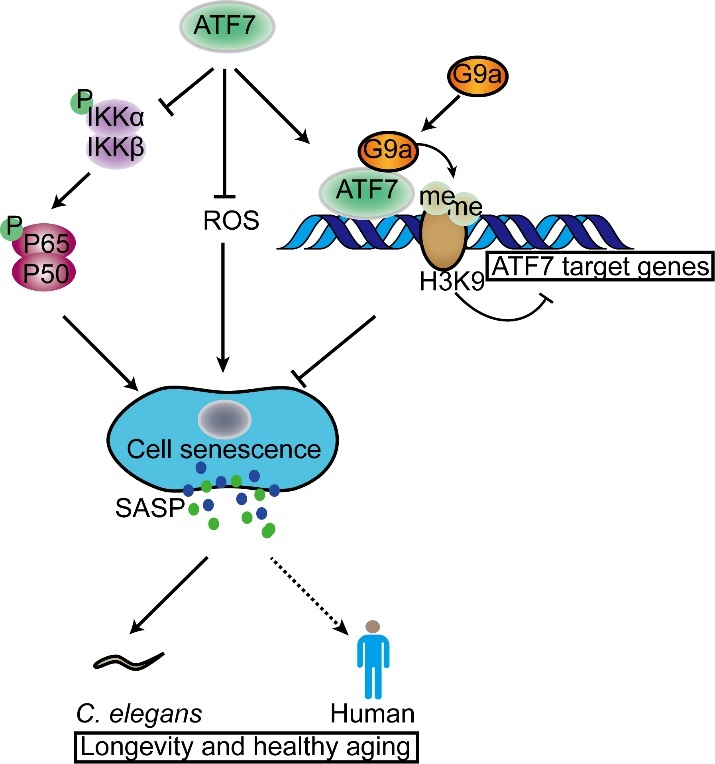
**Schematic role of *ATF7* in senescence process**. *ATF7* influences senescence via three pathways. First, *ATF7* represses activation of NF-κB signaling. Second, *ATF7* silences target genes, including SASP-related genes, by recruiting histone H3K9 dimethyltransferase G9a. These two pathways synergistically decrease SASP factor production. Third, *ATF7* directly inhibits ROS production, thus repressing senescence. These pathways may synergistically lead to lifespan extension in *C. elegans* and humans.

Cellular senescence can be induced by various stimuli, including DNA damage, oncogene activation, oxidative stress, chemotherapy, mitochondrial dysfunction, and epigenetic changes [78]. Our data showed that *ATF7*-dependent senescence may be mediated through its regulation of oxidative stress and epigenetic changes (Fig. 6). SASP factors are primarily regulated by transcription [79]. Upstream regulators of SASP, such as p38MAPK, JAK2/3, TGF-β, and PI3K-Akt, converge into two transcriptional mechanisms, i.e., the NF-κB pathway and C/EBP family [6]. While our results showed that *ATF7* significantly represses NF-κB activity, whether C/EBP is involved remains to be investigated.

ATF7 is also reported to suppress a subset of genes associated with innate immunity by recruiting the H3K9 dimethyltransferase G9a [18]. For example, high H3K9me2 levels around the promoters of SASP factors *IL6* and *IL8* have been shown to decrease their induction [58]. Our data indicated that overexpression of *ATF7* markedly increased H3K9me2 levels, and ATF7 directly bound to and recruited G9a to dimethylate H3K9, further suppressing genes encoding SASP factors (Fig. 6). These findings suggest that *ATF7* plays a role in delaying cellular senescence and inhibiting SASP production, which may function to regulate individual aging. Furthermore, our results showed that overexpression and depletion of *ATF7* delayed and accelerated aging, and extended and shortened *C. elegans* lifespan, respectively. However, the exact mechanism by which *ATF7* protects *C. elegans* from aging has not yet been determined as the immune system of *C. elegans* differs from that of humans, although preliminary RNA-seq data suggest that innate immune functions may be involved. Taken together, our results suggest that *ATF7* plays an important role in promoting healthy aging and longevity by regulating cellular senescence and inflammatory genes (Fig. 6).

In conclusion, we identified an up-regulation of transcription factor *ATF7* in LLIs and demonstrated its role in promoting longevity in a model organism. *ATF7* inhibited senescence and SASP factors by suppressing NF-κB pathway activity and increasing H3K9 dimethylation, suggesting that it may inhibit persistent systemic chronic inflammation and promote lifespan in LLIs. However, further research is necessary to determine whether *ATF7* may be a target for reducing age-related pathology associated with chronic inflammation.

## Supplementary Materials

The Supplementary data can be found online at: www.aginganddisease.org/EN/10.14336/AD.2022.1217.
