# Supplementary material for: Longevity-Associated Transcription Factor ATF7 Promotes Healthspan by Suppressing Cellular Senescence and Systematic Inflammation
Source: Aging Dis. 2023 Aug 1;14(4):1374–89. doi: 10.14336/AD.2022.1217 (PMC10389835; doi:10.14336/AD.2022.1217)
Supplement: Supplementary file 1 — The Supplementary data can be found online at: www.aginganddisease.org/EN/10.14336/AD.2022.1217. [file AD-14-4-1374-s.pdf]

SUPPLEMENTARY DATA

Longevity-Associated Transcription Factor *ATF7* Promotes Healthspan by Suppressing Cellular Senescence and Systematic Inflammation

Yaqun Huang, Ming-Xia Ge, Yu-Hong Li, Jing-Lin Li, Qin Yu, Fu-Hui Xiao, Hong-Shun Ao, Li-Qin Yang, Ji Li, Yonghan He, Qing-Peng Kong

© 2022. Huang Y et al. Published online at https://www.aginganddisease.org/EN/10.14336/AD.2022.1217

SUPPLEMENTARY DATA

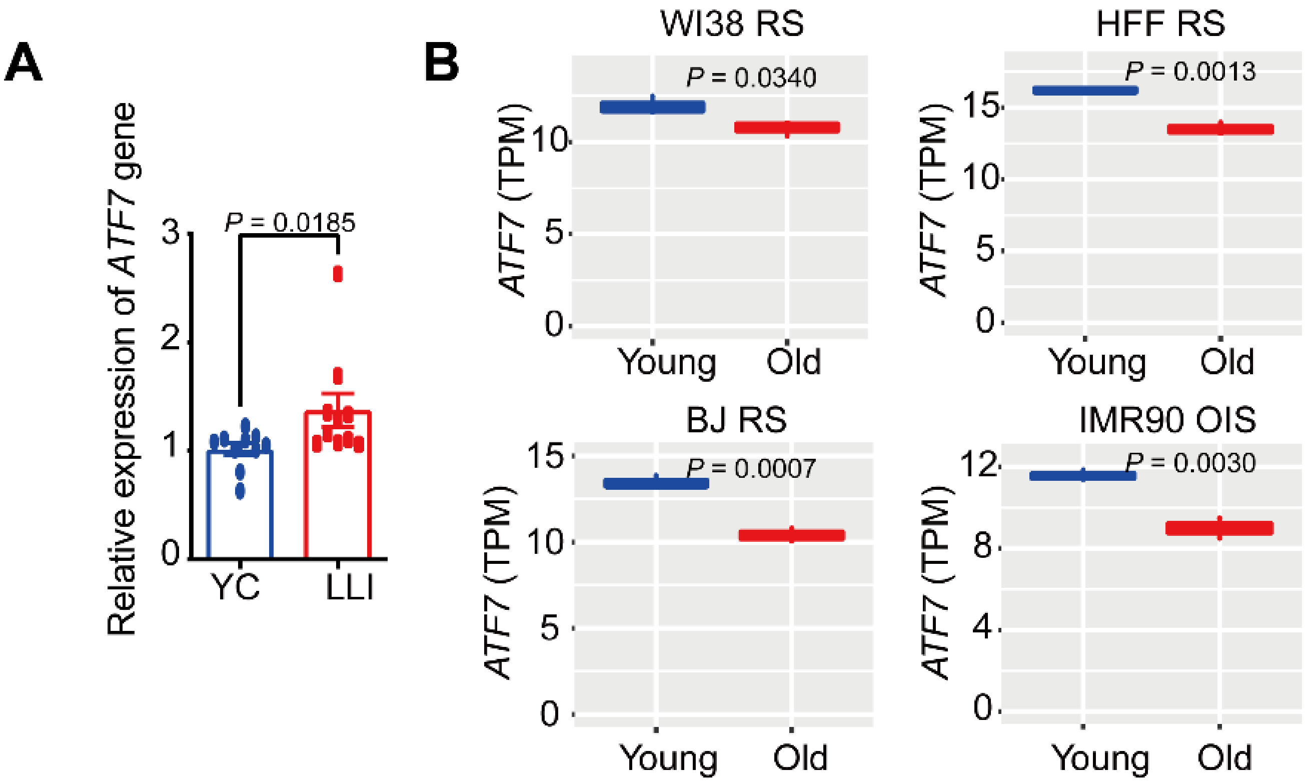

Supplementary Figure 1. *ATF7* expression is up-regulated in LLIs, while down-regulated in senescent HDFs. (A) qRT-PCR of different *ATF7* expression levels between LLIs and YCs (n = 10 biological replicates/group). (B) *ATF7* mRNA levels were assessed in senescent WI-38 fibroblasts, HFF and BJ fibroblasts, and IMR-90 fibroblasts compared to non-senescent cells. LLIs: long-lived individuals; YCs: younger controls. Mann-Whitney U test for A.

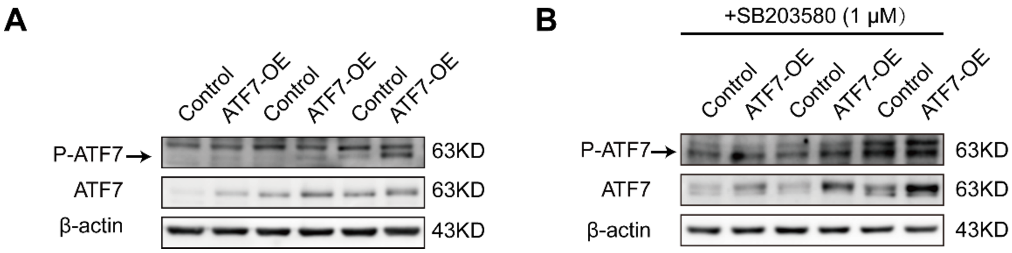

Supplementary Figure 2. ATF7 overexpression efficiency decreases during serial passage in HDFs. (A) ATF7 overexpression efficiency and phosphorylation during serial passage in control and *ATF7*-overexpressing HDFs by western blotting (n = 2 biological replicates). (B) ATF7 overexpression efficiency and phosphorylation during serial passage by western blotting in control and *ATF7*-overexpressing HDFs incubated with p38MAPK inhibitor SB203580 (1 μM) (n = 1 biological replicate). OE, overexpression.

© 2022. Huang Y et al. Published online at https://www.aginganddisease.org/EN/10.14336/AD.2022.1217

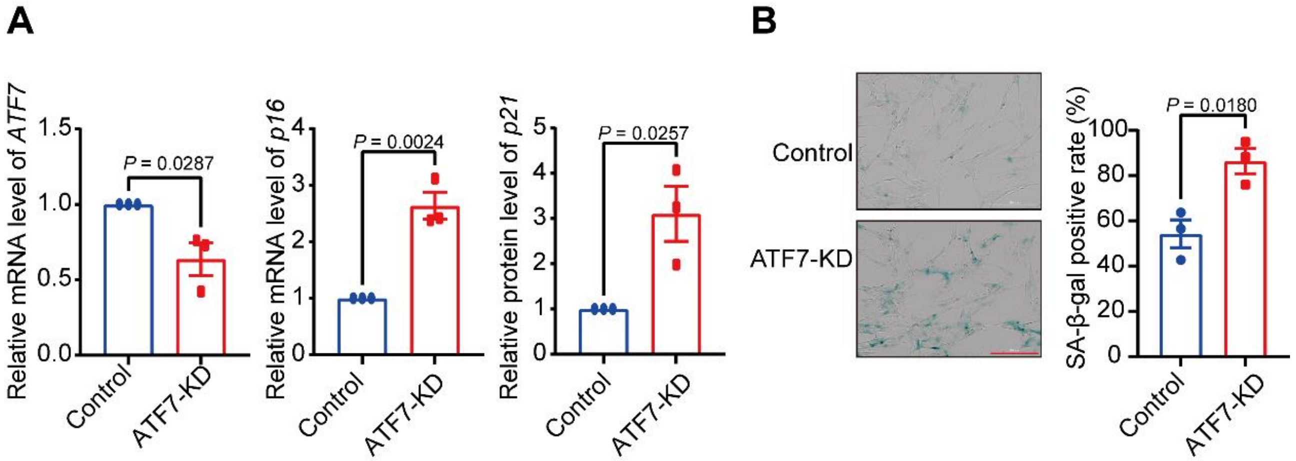

Supplementary Figure 3. Manipulation of *ATF7* expression alters cellular senescence progression in IMR-90 cells. (A) qRT-PCR analysis of mRNA levels of *ATF7*, *p21*, and *p16* in control and *ATF7*-knockdown IMR-90 cells (n = 3 biological replicates). (B) SA-β-Gal-staining cells in control and *ATF7*-knockdown IMR-90 cells. Typical images are presented on the left and quantitative result is presented on the right (n = 3 biological replicates, scale bar, 200 μm). KD, knockdown. Statistical analyses were performed using two-sided Student's *t*-test.

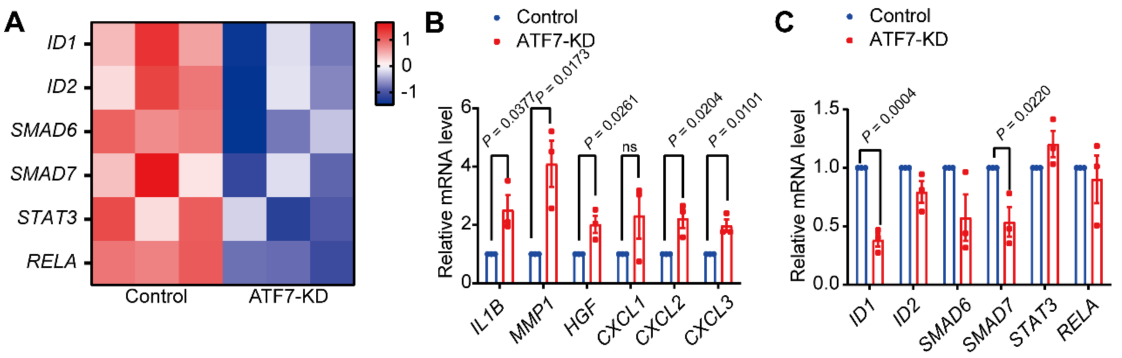

Supplementary Figure 4. Depletion of *ATF7* promotes expression of several SASP factors in HDFs. (A) Heatmap of inflammatory pathway genes in *ATF7*-knockdown HDFs relative to the control from RNA-seq datasets (n = 3 biological replicates). (B-C) qRT-PCR analysis of mRNA levels in SASP genes (B) and inflammatory pathway genes (C) in *ATF7*-knockdown HDFs relative to the control (n = 3 biological replicates). KD, knockdown. Statistical analyses were performed using two-sided Student's *t*-test.

© 2022. Huang Y et al. Published online at https://www.aginganddisease.org/EN/10.14336/AD.2022.1217

SUPPLEMENTARY DATA

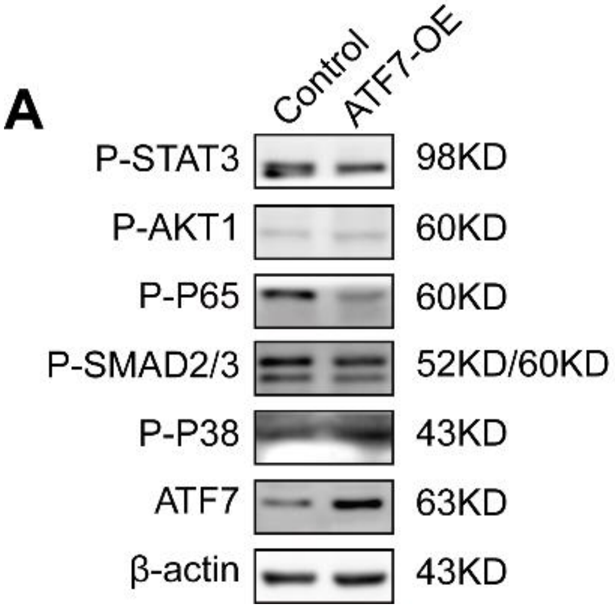

Supplementary Figure 5. *ATF7* inhibits SASP by inhibiting NF-κB signaling in HDFs and recruiting G9a in HEL cells. (A) Inflammatory pathway activation was assessed by western blotting in control and *ATF7*-overexpressing HDFs. (n = 3 biological replicates). (B) Co-immunoprecipitation of ATF7 and G9a. Cell lysates of HEL fibroblasts were immunoprecipitated with anti-ATF7 antibody or control IgG, with immunocomplexes then subjected to western blotting using G9a (n = 2 biological replicates). OE, overexpression.

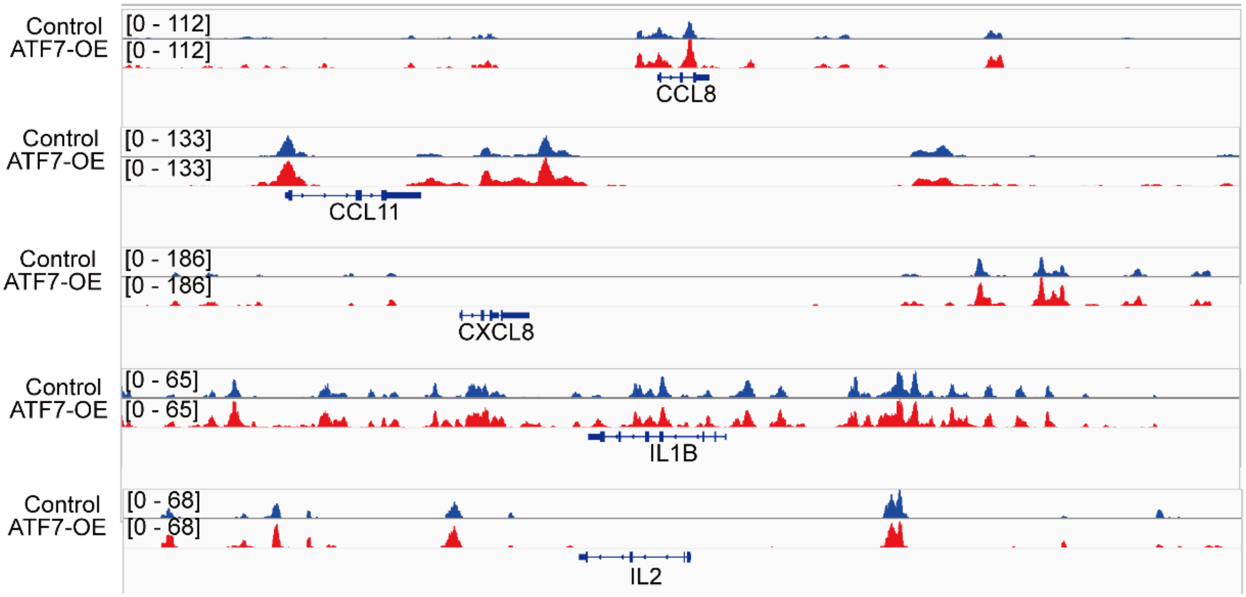

Supplementary Figure 6. *ATF7* inhibits inflammatory genes by modulating H3K9me2 in HDFs, based on CUT&Tag. IGV analysis of H3K9me2 peaks in inflammatory genes in control and *ATF7*-overexpressing cells. (n = 2 biological replicates). OE, overexpression.

© 2022. Huang Y et al. Published online at https://www.aginganddisease.org/EN/10.14336/AD.2022.1217

SUPPLEMENTARY DATA

Supplementary Table 1. Primers used in qRT-PCR analysis

**Name**	**Forward primer**	**Reverse primer**
Human *ATF7*	GAGACGACAGACCGTTTGTGT	AGGCGTTTGATCTGCAATGAT
Human *p16*	GAGCAGCATGGAGCCTTC	CCGCTGCAGACCCTCTAC
Human *p21*	TGTCCGTCAGAACCCATGC	AAAGTCGAAGTTCCATCGCTC
Human *IL1B*	AGCTACGAATCTCCGACCAC	CGTTATCCCATGTGTCGAAGAA
Human *IL6*	ACTCACCTCTTCAGAACGAATTG	CCATCTTTGGAAGGTTCAGGTTG
Human *MMP1*	CTCTGGAGTAATGTCACACCTCT	TGTTGGTCCACCTTTCATCTTC
Human *CCL2*	CAGCCAGATGCAATCAATGCC	TGGAATCCTGAACCCACTTCT
Human *CCL8*	CTTGCTCAGCCAGATTCAGTT	GACCCATCTCTCCTTGGGGT
Human *CXCL1*	CAGGGAATTCACCCCAAGAACA	GGATGCAGGATTGAGGCAAGC
Human *CXCL2*	GAAAGCTTGTCTCAACCCCG	TGGTCAGTTGGATTTGCCATTTT
Human *CXCL3*	AAACCGAAGTCATAGCCACA	ATTTTCAGCTCTGGTAAGGGCA
Human *IGFBP4*	ACTTCCACCCCAAGCAGTGTC	AAGCTTCACCCCCGTCTTC
Human *IGFBP5*	ACCTGAGATGAGACAGGAGTC	GTAGAATCCTTTGCGGTCACAA
Human *IGFBP6*	GAGGGGCTCAAACACTCTACG	CCATCCGATCCACACACCA
Human *HGF*	GACGCAGCTACAAGGGAACA	GCTCGAAGGCAAAAAGCTGTG
Human *FGF7*	GAGCGACACACAAGAAGT	AATTCCAACTGCCACTGT
Human *IL8*	AGTTTTTGAAGAGGGCTGAGA	TGCTTGAAGTTTCACTGGCATC
Human *GM-CSF*	AATGTTTGACCTCCAGGAGCC	AGTGCTGCTTGTAGTGGCTG
Human *ID1*	ACGACATGAACGGCTGTTACTC	GCTCCAACTGAAGGTCCCTGAT
Human *ID2*	ATCCTGTCCTTGCAGGCTTC	ACCGCTTATTCAGCCACACA
Human *SMAD6*	CCTCCCTACTCTCGGCTGTC	GGTAGCCTCCGTTTCAGTGTA
Human *SMAD7*	TCACCTTAGCCGACTCTGCG	TCCAGAAGAAGTTGGGAATCTGA
Human *STAT3*	CAGCAGCTTGACACACGGTA	AAACACCAAAGTGGCATGTGA
Human *NFKB2*	ATGGAGAGTTGCTACAACCCA	CTGTTCCACGATCACCAGGTA
Human *RELB*	CCATTGAGCGGAAGATTCAACT	CTGCTGGTCCCGATATGAGG
Human *ACTB*	AGAGCTACGAGCTGCCTGAC	AGCACTGTGTTGGCGTACAG
C. elegans ATF7	TTGTTATCTGTGGCCGGTGA	ATGGAGAACGAAGGAGTGCC
C. elegans act-1	GCTGGACGTGATCTTACTGATTACC	GTAGCAGAGCTTCTCCTTGATGTC

© 2022. Huang Y et al. Published online at https://www.aginganddisease.org/EN/10.14336/AD.2022.1217

SUPPLEMENTARY DATA

Supplementary Table 2. List of antibodies used in this study

Antibodies	Source	Identifier	Experiment
Actin	Beyotime	AA128-1	Immunoblotting
P16	Proteintech	10883-1-AP	Immunoblotting
ATF7	Abcam	ab87844	Immunoblotting/Co-IP
H3k9me2	Millipore	07-441	Immunoblotting
H3k9me2	Cell Signaling Technology	4658	Immunoblotting/ChIP/CUT&Tag
NF-κB Pathway Antibody Sampler Kit	Cell Signaling Technology	9936	Immunoblotting
G9a	Abcam	Ab185050	Immunoblotting
P-ATF7/ATF2	Cell Signaling Technology	40749	Immunoblotting
P-STAT3	ABclonal	AP0715	Immunoblotting
P-AKT	ABclonal	AP0637	Immunoblotting
P-P65	ABclonal	AP0124	Immunoblotting
P-SMAD2/SMAD3	ABclonal	AP0548	Immunoblotting
P-P38	ABclonal	AP1165	Immunoblotting
Normal rabbit IgG	Cell Signaling Technology	2729	ChIP/CUT&Tag/Co-IP
H3	Cell Signaling Technology	4620	ChIP/CUT&Tag
Anti-Rabbit IgG-HRP	Beyotime	A0208	Immunoblotting
Anti-Mouse IgG-HRP	Beyotime	A0216	Immunoblotting

Supplementary Table 3. Primers used in ChIP-qRT-PCR analysis.

**Name**	**Forward primer**	**Reverse primer**
IL6	CAGCCATCCTCCCCCATTTT	ATTTCAGGACCCGCCTGTTG
IL8	TGATGACTCAGGTTTGCCCT	AGTGCTCCGGTGGCTTTTTA
p16	TGAATCGGGGTGTTTGGTGT	ATTTCGATTCTCGGTGGGGC
CCL8	GGTTTGCCCTGAGGGGATG	ACAGAGCTGCAGAAATCAGGAAGGCT
CXCL1	CTCCAGCCACAAATCCGAGA	GGCGGATCCCTGAGAACCA
CXCL2	AGTTCGGAAGGAAGGCGATG	CAAGACAGTCAGACCCGGAC
CXCL3	TGGTTGAGACTGGAAAGCCC	CCTACCCGTATCCGACTCCA
TNF	GTCACTCATTGCTGAGCCTCT	AGCTTCTTCCCACCCACAAG
STAT1	CGCAGGAAAGCGAAACTACC	CGCAGGAAAGCGAAACTACC

Supplementary Table 4. The suggested roles of upregulated DEG orthologs in humans.

**Gene (*C. elegans*)**	**Orthologs in *Homo sapiens***	**Potential role in human inflammation**	**Reference**
GST-13	*HPGDS*	Pro- or anti-inflammation	[80]
ASP-12	*REN*	Pro- or anti-inflammation	[81]
K12H4.7	*PRSS16*	Anti-inflammation	[82]
ASP-14	*CTSE*	Proinflammation	[83]
PGP-3	*ABCB1*	Anti-inflammation	[84, 85]
CLEC-86	*REG1A*	Anti-inflammation	[86]
LEC-11	*LGALS4*	Pro- or anti-inflammation	[87]
NHR-115	*RORA*	Anti-inflammation	[88]

© 2022. Huang Y et al. Published online at https://www.aginganddisease.org/EN/10.14336/AD.2022.1217
